# Analysis of the role of the low threshold currents I_T_ and I_h_ in intrinsic delta oscillations of thalamocortical neurons

**DOI:** 10.3389/fncom.2015.00052

**Published:** 2015-05-07

**Authors:** Yimy Amarillo, Germán Mato, Marcela S. Nadal

**Affiliations:** ^1^Consejo Nacional de Investigaciones Científicas y Técnicas, Física Estadística e Interdisciplinaria, Centro Atómico BarilocheSan Carlos de Bariloche, Argentina; ^2^Comisión Nacional de Energía Atómica and Consejo Nacional de Investigaciones Científicas y Técnicas, Centro Atómico Bariloche and Instituto BalseiroSan Carlos de Bariloche, Argentina

**Keywords:** T-type calcium channel, thalamocortical neurons, repetitive burst firing, sub-threshold conductances

## Abstract

Thalamocortical neurons are involved in the generation and maintenance of brain rhythms associated with global functional states. The repetitive burst firing of TC neurons at delta frequencies (1–4 Hz) has been linked to the oscillations recorded during deep sleep and during episodes of absence seizures. To get insight into the biophysical properties that are the basis for intrinsic delta oscillations in these neurons, we performed a bifurcation analysis of a minimal conductance-based thalamocortical neuron model including only the I_T_ channel and the sodium and potassium leak channels. This analysis unveils the dynamics of repetitive burst firing of TC neurons, and describes how the interplay between the amplifying variable m_T_ and the recovering variable h_T_ of the calcium channel I_T_ is sufficient to generate low threshold oscillations in the delta band. We also explored the role of the hyperpolarization activated cationic current I_h_ in this reduced model and determine that, albeit not required, I_h_ amplifies and stabilizes the oscillation.

## Introduction

Repetitive burst firing of thalamocortical (TC) neurons in the delta band has been linked to the expression of the rhythms that characterize slow wave sleep and the pathological spike and wave discharges of absence epilepsy (McCormick and Bal, 1997; Budde et al., 2005). The synchronized expression of repetitive bursting in TC neurons of the behaving animal is the result of the interaction between the intrinsic properties of these neurons and the synaptic activity of the thalamo-reticulo-cortical network (Lytton et al., 1996; Destexhe and Sejnowski, 2003). The prominent role of intrinsic ionic mechanisms in the generation and maintenance of the oscillations at the cellular level has been extensively demonstrated: individual TC neurons maintained *in vitro* are able to fire bursts repetitively either spontaneously or after injection of hyperpolarizing current, and this ability is indeed conserved under conditions of synaptic isolation (McCormick and Pape, 1990; Leresche et al., 1991). In addition, the EEG expression of rhythms associated with repetitive burst firing of TC neurons (slow wave oscillations during deep sleep and spike and wave discharges during absence seizures) are strongly affected by genetic or pharmacological manipulation of the ion channels expressed by TC neurons (Kim et al., 2001; Ludwig et al., 2003; Lee et al., 2004; Anderson et al., 2005; Budde et al., 2005). Specifically, genetic elimination of the calcium channel pore forming subunit CaV3.1 (the main channel subunit carrying I_T_ in TC neurons from mice) abolishes the generation of low threshold spikes (LTS) (Kim et al., 2001), and suppresses the delta oscillations during NREM sleep (Lee et al., 2004). Conversely, overexpression of this channel subunit results in a phenotype of pure absence epilepsy in mice (Ernst et al., 2009). An absence epilepsy phenotype is also obtained by genetic elimination of HCN2 (Ludwig et al., 2003), which is an I_h_ channel subunit strongly expressed in TC neurons (Santoro et al., 2000).

It has been previously shown that the two firing regimes of thalamocortical neurons, tonic and burst firing, can be described by two distinct oscillatory systems that operate independently at different membrane potentials and at different time scales (Rush and Rinzel, 1994). In a previous study, we characterized the seven different conductance's that operate at hyperpolarized membrane potentials, and established their contributions to intrinsic delta oscillations. In that study, we showed that the minimal ionic mechanisms required for generation and maintenance of oscillations compatible with physiological (or pathological) repetitive burst firing are I_T_ and the leak currents (Amarillo et al., 2014). Here we analyze the bifurcation structure of this minimal model and show that the system can enter the oscillatory regime (limit cycle) from two different stable equilibriums (which occur at physiologically plausible membrane potentials) via different dynamic mechanisms: the transition from a depolarized stable equilibrium occurs via a supercritical Hopf bifurcation, whereas at hyperpolarized potentials the system can undergo either a subcritical Hopf bifurcation or a saddle-node bifurcation on invariant cycle (Izhikevich, 2005). We discuss possible functional, physiological and pathophysiological implications of this dynamic behavior.

Although a role of I_h_ in repetitive burst firing of TC neurons has been suggested previously (McCormick and Pape, 1990; Soltesz et al., 1991; Hughes et al., 1998), our simulations indicate that I_h_ is not essential for repetitive burst firing (Amarillo et al., 2014). It has been previously demonstrated that the main role of I_h_ is the stabilization of the resting membrane potential (RMP), and that this current is one of the main determinants of the positive shift of the RMP from the potassium equilibrium potential in TC neurons (Amarillo et al., 2014 and references therein). In this study, we use phase plane and bifurcation analysis to determine the role of Ih in repetitive burst firing and we show that I_h_ stabilizes the oscillations by increasing the voltage range (and the range of current injection magnitudes) at which stable oscillations occur. Some of these results have been presented previously in abstract form (Amarillo and Nadal, 2013).

## Methods

The HH-like equations used in this study have been published elsewhere (Amarillo et al., 2014). Briefly, the voltage equations for the minimal I_T_-Leaks model and the I_T_-I_h_-Leaks model are:

(1) $$dV/dt=(I_{inj}-I_{T}-I_{Kleak}-I_{Naleak})/C$$

and

(2) $$dV/dt=(I_{inj}-I_{T}-I_{h}-I_{Kleak}-I_{Naleak})/C$$

respectively; where *I_Kleak_* = *g_Kleak_* (*V* − *E_Kleak_*)*S*, *I_Naleak_* = *g_Naleak_* (*V* − *E_Naleak_*)*S* are the leak currents, *I_inj_* is the injected current, *C* is the membrane capacitance and *S* the surface of the neuron (see parameter values in Tables 1, 2).

**Table 1:** **Model cell and ion channel parameters**.

	*C* = 0.2 *nF*
	*S* = 20000 μ*m*^2^
	*T* = 36°*C*
	*Ca_o_* = 2 × 10^−3^*M*
	*Ca_i_* = 0.05 × 10^−6^*M*
I_Kleak_	*g_Kleak_* = 1 × 10^−5^*S/cm*^2^ *E_Kleak_* = −100*mV*
I_Naleak_	*g_Naleak_* = 3 × 10^−6^*S/cm*^2^ *E_Naleak_* = 0 *mV*
I^(a)^_T_	*m*_*T*∞_(*V*) = 1/(1 + *exp*[(*V* − ***V_1/2mT_***/−6.2]) τ*_mT_*(*V*) = (0.612 + 1/(*exp*[(*V* − ***V*_τ*m1*_**)/−16.7] + *exp*[(*V* − ***V*_τ*m2*_**)/18.2]))/3 *h*_*T*∞_(*V*) = 1/(1 + *exp*[(*V* − ***V*_*1/2hT*_**)/4]) τ_*hT*_(*V*) = (*exp*[(*V* − ***V*_τ*h1*_**)/66.6])/3 *for V* < −75 mV τ*_hT_*(*V*) = (28 + *exp*[(*V* − ***V*_τ*h2*_**)/−10.5])/3 *for V* > −75*mV* ***p_T_*** = *see* Table 2.
I_h_	*m*_*h*∞_(*V*) = 1/(1 + *exp*[(*V* + 82)/5.49]) τ*_mh_*(*V*) = (1/([0.0008 + 0.0000035 *exp*(−0.05787*V*)] + *exp*(−1.87 + 0.0701 V)))/1.32 *g_h_* = 2.2 × 10^−5^*S/cm*^2^ *E_h_* = −43*mV*

*^(a)^Parameters that are changed in the different conditions considered in the paper are shown in bold (see specific values in Table 2)*.

**Table 2:** **Maximum permeability and voltage dependence parameters of I_T_**.

**Condition**	***p_T_* (*cm/s*)**	***V*_1/2*mT*_(*mV*)**	***V*_τ*m*1_(*mV*)**	***V*_τ*m*2_(*mV*)**	***V*_1/2*hT*_(*mV*)**	***V*_τ*h*1_(*mV*)**	***V*_τ*h*2_(*mV*)**	**Figures**
Default 3D	7.0 × 10^−5^	−53	−128	−12.8	−75	−461	−16	1A, 1B, 1E^*^, 3, 4
Default 2D	7.0 × 10^−5^	−53	–	–	−75	−461	−16	1A, 2
Activation shifted −3 mV	3.0 × 10^−5^	−56	−131	−15.8	−75	−461	−16	1C, 1F^*^
McCormick and Huguenard	1.1 × 10^−4^	−57	−132	−16.8	−81	−467	−22	1D

*Global voltage shifts are applied on all voltage dependence parameters of a given gating variable of I_T_ as previously performed (Destexhe et al., 1998; Amarillo et al., 2014)*.

**Figure 1:**
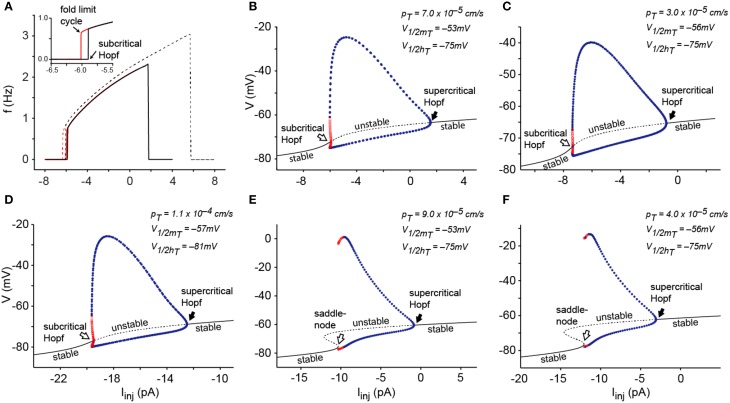
**Dynamical mechanisms of oscillations in the I_T_-Leaks model**. **(A)** Frequency-current plots of oscillations induced by sustained injection of depolarizing current from a hyperpolarized stable potential (black) and injection of hyperpolarizing current from a depolarized stable potential (red) for the 3D I_T_-Leaks model (solid lines) and for the 2D I_T_-Leaks model (dashed lines). The inset shows magnification of the region of mismatch (hysteresis) between the two plots at the lowest frequencies for the 3D model. **(B)** Bifurcation diagram of the I_T_-Leaks model using the default value of *p_T_* (7.0 × 10^−5^ cm/s) while maintaining other parameters at default. The diagram shows voltage at fixed points (black V/I curve) and max/min of limit cycle as the current injection is changed (dots). The dashed region on the V/I curve (black) and the red dots represent instability whereas the continuous line and blue dots represent regions of stable equilibrium and stable periodic orbits respectively. **(C)** Bifurcation diagram after shifting the activation variable of I_T_ by −3 mV, using *p_T_* = 3.0 × 10^−5^ cm/s. **(D)** Bifurcation diagram of the I_T_-leaks model with kinetic parameters and voltage dependence as in McCormick and Huguenard (1992). *p_T_* set to 11 × 10^−4^ cm/s. **(E)** Bifurcation diagram of the I_T_-leaks model using a *p_T_* value of 9.0 × 10^−5^ cm/s while maintain other parameters at default. **(F)** Bifurcation diagram after shifting m_T_ by −3 mV, using a *p_T_* value of 4.0 × 10^−5^ cm/s. Conventions for panels **(C–F)** as in **(B)**.

* *In Figures 1E,F the parameters of voltage dependence are as indicated in the table, however the values of p_T_ are 9 × 10 ^−5^ and 4 × 10^−5^ cm/s respectively (see text)*.

The equations used for I_T_ are:

(3) $$I_{T}=p_{T}m_{T}^{2}h_{T}SG(V,Ca_{o},Ca_{i})$$

(4) $$dm_{T}/dt=(m_{T\infty }(V)-m_{T})/\tau_{mT}(V)$$

(5) $$dh_{T}/dt=(h_{T\infty }(V)-h_{T})/\tau_{hT}(V),$$

where *p_T_* is the maximum permeability, *m_T_* and *h_T_* are the activation and inactivation variables respectively and *m*_*T*∞_(*V*), *h*_*T*∞_(*V*), τ*_mT_*(*V*), τ_*hT*_(*V*) are the steady state and time constants of activation and inactivation (see Tables 1, 2). *G*(*V, Ca_o_,Ca_i_*) is a non-linear function of potential and calcium concentration;

(6) $$\begin{array}{l} G(V,Ca_{o},Ca_{i})=z^{2}F^{2}V/RT(Ca_{i}-Ca_{o}exp[-zFV/RT])/\\ \;(1-exp[-zFV/RT]), \end{array}$$

where *Ca_o_* and *Ca_i_* are the extracellular and the intracellular concentrations of Ca^++^ and *z*, *F*, *R*, and *T* are the valence, the Faraday constant, the gas constant and the absolute temperature respectively.

The equations for I_h_ are:

(7) $$I_{h}={\bar{g}}_{h}m_{h}\left(V-E_{h}\right)S$$

(8) $$dm_{h}/dt=(m_{h\infty }(V)-m_{h})/\tau_{mh}(V)$$

where *g_h_* is the maximum conductance, *m_h_* is the activation variable, *E_h_* is the reversal potential and *m*_*h*∞_(*V*) and τ*_mh_*(*V*) are the steady state activation and time constant respectively (see Table 1).

Bifurcation analysis and phase plane portrait analysis were performed using XPPAUT (Ermentrout, 2002). In order to convert the three-dimensional I_T_-leaks model containing three differential equations (*dV/dt*, *dm_T_/dt*, and *dh_T_/dt*) into a two-dimensional model (containing only *dV/dt* and *dh_T_/dt*), we assumed an instantaneous activation of I_T_ and replaced the time dependent equation for the steady state equation of the *m* variable of I_T_. Thus, the current Equation (3) was replaced by

(9) $$I_{T}=p_{T}m_{T\infty }^{2}(V)h_{T}SG(V,Ca_{o},Ca_{i})$$

Frequency current plots (Figure 1A) were obtained by averaging the inter LTS intervals occurring during each of 4000 current steps of 10 s, between −10 and +10 pA, using a second order Runge-Kutta integration algorithm with a 0.01 ms time step (Press et al., 1992).

## Results

### Phase plane and bifurcation analysis of a minimal model of thalamocortical neurons

In a previous study, we used a murine TC neuron conductance model to explore the minimal requirements for generating periodic oscillations at delta frequencies (Amarillo et al., 2014). In the minimal model, which only has the calcium current I_T_ and the leak currents (*I_Kleak_* and *I_Naleak_*), the propensity to fire repetitive bursts is strongly affected by the permeability of the I_T_ current. The baseline value of maximum permeability of I_T_ in this minimal model corresponds to the experimental value obtained from rodent TC neurons *in vitro*: *p_T_* = 5 × 10^−5^ cm/s. In agreement with the rodent experimental data, the TC model has a low propensity to fire repetitive bursts when using this baseline *p_T_* value of I_T_. However, increasing the availability of I_T_—by manipulating its maximum permeability or its voltage dependence of activation and inactivation—enables periodic low threshold oscillations. Thus, while keeping the value of *p_T_* at 5 × 10^−5^ cm/s, changes in the gating variables of I_T_ that result in a larger window current component (a global hyperpolarizing shift larger than −2 mV in the activation variable *m_T_*, or a global depolarizing shift larger than +2 mV of the inactivation variable *h_T_*) favor sustained oscillations (see Figure 8 in Amarillo et al., 2014). Similarly, increasing the maximum permeability *p_T_* by less than 30% (from 5 to 7 × 10^−5^ cm/s) induces spontaneous oscillations of 32 mV of amplitude at 2.3 Hz. We take this value (7 × 10^−5^ cm/s) as default in the rest of this paper.

Here we analyze the transitions from two stable equilibriums, one occurring at a relatively depolarized membrane potential *V* (positive to about −63 mV) and the other occurring at hyperpolarized *V* (negative to about −75 mV) into a stable limit cycle by using injected current *I_inj_* as parameter. We first analyzed the bifurcation structure of the 3-dimentional system comprising the differential Equations (1), (4), and (5). Figure 1A shows frequency current plots of the system using the default value of *p_T_*. These plots reveal a small range of bistability (hysteresis, see inset) which is bounded in the left side by a fold limit cycle bifurcation and on the right side by a subcritical Hopf bifurcation. In this bifurcation, the stable limit cycle coalesces with an unstable limit cycle and both disappear. We also compared the 3D dynamical system with a 2D system obtained by making instantaneous the kinetics of the *m* gate of I_T_ (see Methods), i.e., making *m_T_* = *m*_*T*∞_(*V*). This is justified because there is a one order of magnitude difference between activation and inactivation time constants (with activation being faster than inactivation). We found that the change of dimensionality from 3D to 2D does not modify the oscillatory activity of the model in response to either injection of hyperpolarizing current from a positive equilibrium or injection of depolarizing current from a negative equilibrium. In fact we found that the bifurcation structure is exactly the same, although the positions of the bifurcations points are changed (Figure 1A).

The bifurcation diagram shows that the transition from the resting state to the limit cycle and from the limit cycle to rest occurs via a *subcritical Hopf* bifurcation at hyperpolarized potentials (Figure 1B, open arrow) whereas the transitions at depolarized voltages occur via a *supercritical Hopf* bifurcation (Figure 1B, filled arrow).

We repeated this analysis using different voltage dependences (global shifts) of the activation and inactivation gates of I_T_ and found the same bifurcation structure. Figure 1C shows the bifurcation diagram of the I_T_-Leaks model after shifting the activation of I_T_ by −3 mV using *p_T_* = 3.0 × 10^−5^ cm/s. Furthermore, the bifurcation diagram of the I_T_-Leaks model using the same values of voltage dependence of activation of I_T_ as in the seminal study by McCormick and Huguenard (1992) (see Table 2), with *p_T_* set to 1.1 × 10^−4^ cm/s, also have the same structure (Figure 1D). This indicates that the dynamical properties of I_T_ are insensitive to small variations of voltage dependence provided that the maximum permeability of I_T_ is kept to the minimum required to enable sustained oscillations. However, setting *p_T_* to slightly higher values (above 8.0 × 10^−5^ cm/s) changes the type of bifurcation occurring at hyperpolarized potentials from a subcritical Hopf to a saddle-node bifurcation on invariant cycle, as the I/V relationship changes from monotonic to non-monotonic (Figure 1E). Similarly, setting *p_T_* = 4.0 × 10^−5^ cm/s after shifting the activation of I_T_ by -3 mV, also changes the type of bifurcation occurring at hyperpolarized potentials from subcritical Hopf to saddle node on invariant cycle (Figure 1F). This dynamical behavior indicates that at some intermediate value of *p_T_* the model should undergo a *Bogdanov-Takens* codimension-2 bifurcation (Izhikevich, 2005). On the other hand the bifurcation occurring at depolarized potentials stays a supercritical Hopf for all values of *p_T_*.

**Figure 2:**
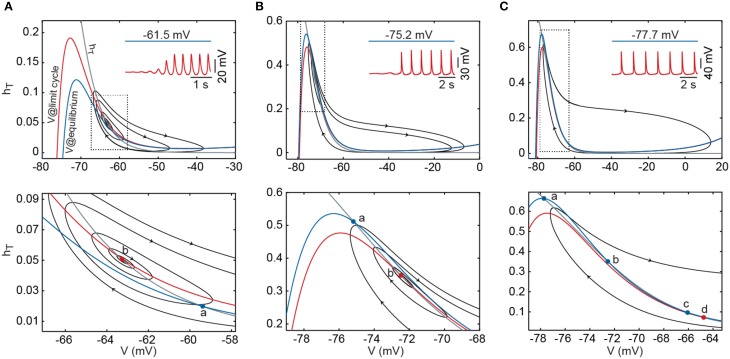
**Phase-plane portrait of the I_T_-leaks model after reduction of dimensionality from 3D to 2D**. **(A)** Using a *p_T_* value of 7.0 × 10^−5^ cm/s, at a depolarized equilibrium (*I_inj_* = 6 pA) the membrane potential is stable at −61.5 mV (blue trace in inset). At this potential, the *V* nullcline (blue line) intersects the *h_T_* nullcline (gray line) on a single point (*a*). Decreasing *I_inj_* to 2 pA induces oscillations (red trace in inset) and in the phase-plane plot, shifts the *V* nullcline upwards (red line) with loss of stability of the intersecting point (*b*) and appearance of a stable limit cycle (supercritical Hopf bifurcation). Black orbits represent the variation of *V* as function of *h_T_* in the direction indicated by the arrow heads. **(B)** Using the same *p_T_* value as in **(A)**, at a hyperpolarized equilibrium (*I_inj_* = −7 pA) the membrane potential stabilized at −75.2 mV (blue trace in inset), the *V* (blue line) and *h_T_*(gray line) nullclines intersect in a single stable point (*a*). Changing *I_inj_* to −6 pA induces oscillations (red trace in inset) and, in the phase plane portrait, shifts the *V* nullcline (red line) downwards with loss of stability of the intersecting point (*b*) and disappearance of an unstable limit cycle (subcritical Hopf bifurcation). **(C)** After increasing *p_T_* to 9.0 × 10^−5^ cm/s, at a hyperpolarized equilibrium (*I_inj_* = −11 pA), the membrane potential is stabilized at −77.7 mV (blue trace in inset). In the phase plane portrait, *V* (blue line) and *h_T_*(gray line) nullclines intersect in three points; one stable point (*a*) and two unstable points (*b* and *c*). At a lower level of hyperpolarization (*I_inj_* = −10 pA), depolarizing current shifts the *V* nullcline (red line) downwards with destruction of the points *a* and *b*, leaving one unstable point (*d*) and allowing the model to oscillate at very low frequencies. Bottom panel shows a magnification corresponding to the encircled area on the upper panel. Conventions for panels **(B,C)** as in **(A)**.

To explore this further, we analyzed the 2D model using a graphic visualization in phase plane portraits. We analyzed the phase plane portraits near the bifurcation points using either the default value of *p_T_* (Figures 2A,B) or a slightly increased value (9.0 × 10^−5^ cm/s, Figure 2C). Figure 2A shows the transition from a depolarized equilibrium to the limit cycle as hyperpolarizing current is injected beyond the threshold for generating repetitive LTSs. At equilibrium, the nullclines intersect at a single stable point (labeled *a*). Injection of hyperpolarizing current displaces the *V* nullcline upwards and destabilizes the intersecting point through a supercritical Hopf bifurcation initiating the oscillation (superimposed orbits). Figure 2B shows the transition from a hyperpolarized equilibrium to the limit cycle after injection of depolarizing current injection. At equilibrium, the nullclines intersect each other at a single stable point. Injection of depolarizing current beyond the LTSs threshold shifts the *V* nullcline downwards; this destabilizes the intersecting point through a subcritical Hopf bifurcation, thereby initiating the oscillation. The same transition is shown in Figure 2C after increasing *p_T_* to 9.0 × 10^−5^ cm/s. In this case, the nullclines intersect at three points at equilibrium. Injection of depolarizing current beyond the LTS threshold shifts the *V* nullcline downwards and cause the destruction of two of the intersecting points (points *a* and *b*), triggering the abrupt appearance of the oscillation. The change of the dynamical behavior from one type of bifurcation to another—that in this case depends both on the magnitude of current injection and on the maximum permeability of I_T_—indicates again that the model can undergo a codimension-2 *Bogdanov-Takens* bifurcation, which is the only possible codimension-2 bifurcation in 2-dimensional systems (Izhikevich, 2005).

### Understanding the role of I_H_ in periodic burst firing

**Figure 3:**
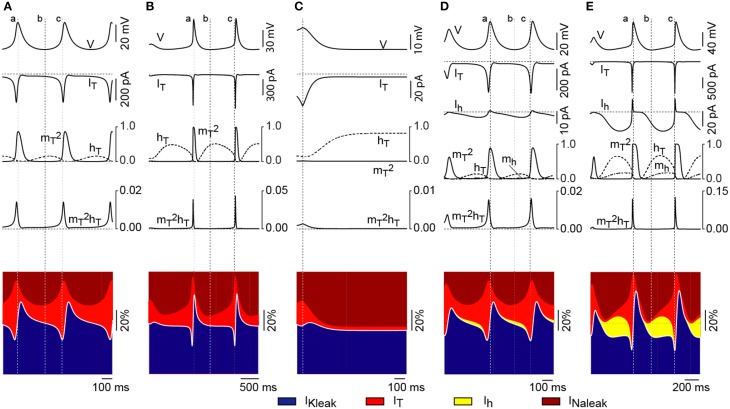
**I_h_ has different effects on the two bifurcations**. **(A)** Upper trace shows the time course of the membrane potential (V) during oscillations elicited at −1 pA (near the upper bifurcation) in the absence of I_h_. The time courses of I_T_ and its gating variables are shown aligned underneath. The bottom diagram shows the time course of the relative contribution of the three currents considered (outward currents in blue and inward currents represented by shades of red and yellow). The white solid line separates total outward from total inward components and therefore represents the net depolarizing or hyperpolarizing drive of the model at a given time point. Vertical dotted lines are positioned at: the peak of the oscillation (a); the valley of the oscillation (b); and the time point of maximum contribution of I_T_ (c). Horizontal dotted lines represent zero values for currents and gating variables and 50% of the total current in diagrams of relative contribution. **(B)** Time course of I_T_, its gating variables and the relative contribution of the currents during oscillations elicited at −4.5 pA (near the lower bifurcation) in the absence of I_h_. **(C)** Time course of I_T_, its gating variables and the relative contribution of the currents for a large hyperpolarizing current injection (without oscillations) in the absence of I_h_. Vertical dotted line is at the onset of current injection. **(D)** Time course of I_T_, I_h_, their gating variables and the relative contribution of the currents during oscillations elicited at −3 pA (near the upper bifurcation) after switching on I_h_. **(E)** Time course of I_T_, I_h_, their gating variables and the relative contribution of the currents during oscillations produced under strong hyperpolarization (−23 pA, near the lower bifurcation) in the presence of I_h_. Conventions in panels **(B–E)** as in panel **(A)**.

We next explored the interaction between I_h_ and I_T_ in the presence of the leak conductances. With *p_T_* set to the default value and I_h_ switched off, oscillations occur in a narrow range of current injection values (approximately between +2 and −6 pA; Figure 1B). With no current or minimal values of current injection (Figure 3A), the availability of I_T_ is small because the inactivation is large (small *h_T_*). Injection of hyperpolarizing current—up to −6 pA—further removes the inactivation of I_T_ and gives rise to oscillations with larger amplitudes (Figure 3B). Under these conditions, oscillations are maintained solely by the regenerative activation of I_T_. Injection of hyperpolarizing current larger than −6 pA induces stronger de-inactivation of I_T_; yet, activation never develops because the drive of the current injection is overpowering (Figure 3C). With I_h_ switched on (Figures 3D,E), the RMP is shifted toward depolarized values due to the steady activation of I_h_. In this case, injection of low values of hyperpolarizing current produces a similar sequence of de-inactivation/activation of I_T_ as in the absence of I_h_ (compare I_T_ gating variables in Figures 3A,D). Notice that both the current magnitude and the gating variables of I_T_ reach similar values with or without little activation of I_h_. In contrast, larger magnitudes of hyperpolarizing current (negative to −6 pA) lead to stronger activation of I_h_, which results in the removal of a larger fraction of inactivation of I_T_ (Figure 3E). Hence, the depolarizing drive contributed by I_h_ not only adds to the regenerative activation of I_T_ during the ascending phase of the LTS, giving rise to larger and faster oscillations, but it also permits the occurrence of these oscillations at much hyperpolarized levels (in contrast to the case shown in Figure 3C). The resulting effect is that I_h_ strongly affects the bifurcation that occurs at hyperpolarized potential yet it affects weakly the bifurcation that takes place at depolarized potential.

**Figure 4:**
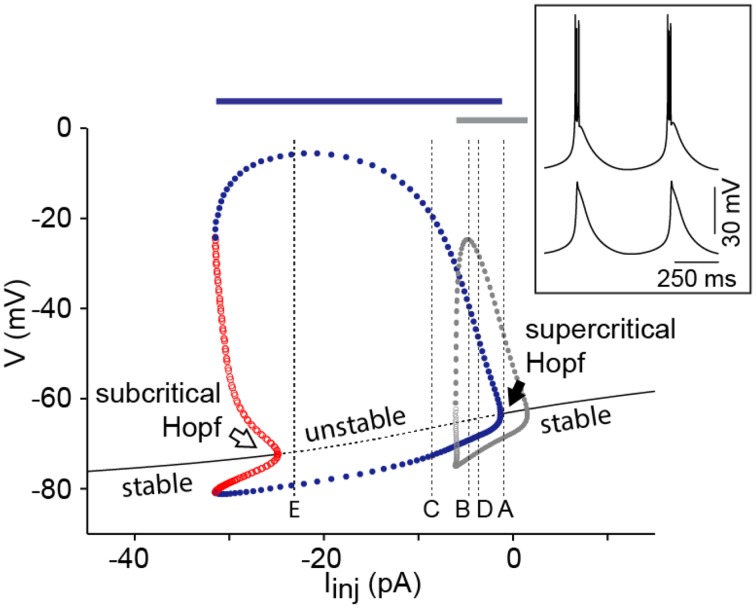
**I_h_ adds robustness to the oscillations**. Bifurcation diagram of the I_T_-I_h_-Leaks model using a *p_T_* value of 7 × 10^−5^ cm/s while maintaining other parameters at default values (see Table 1 and Amarillo et al., 2014). Superimposed in gray is the max/min of limit cycle of the I_T_-leaks model (see Figure 1B). Vertical dotted lines labeled **(A–E)** are positioned at the values of current injection used in the corresponding panels of Figure 3. Conventions are as in Figure 1. The inset shows an example of the repetitive bursting oscillations elicited in the presence of I_h_ and the spiking mechanisms (I_Na_ and I_K_) using *I_inj_* = −8 pA (upper trace) and oscillations elicited in the presence of Ih and absence of spiking mechanisms using the same value of *I_inj_* (lower trace). The bars above the bifurcation diagram indicate the range of *I_inj_* that elicits oscillations in the model with spiking mechanisms both in the presence of I_h_ (blue bar) and in the absence of I_h_ (gray bar).

The bifurcation diagram of this four-dimensional system [with differential Equations (2), (4), (5) and (8)] shows an I/V relationship that is monotonic for a large range of *p_T_* values. In order to change the I/V relationship to non-monotonic under these conditions, *p_T_* has to be increased above 1.5 × 10^−4^ cm/s. This does not imply, however, that a saddle-node bifurcation takes place because for the values of the current where the I/V curve becomes non-monotonic, the resting state has already lost stability via a Hopf bifurcation (data not shown). The diagram shows that the system enters the limit cycle via a supercritical Hopf bifurcation at depolarized potentials, and via a subcritical Hopf at hyperpolarized potentials (Figure 4). The bifurcation diagram also shows that the range of current injection that elicits oscillations is much broader in the presence of I_h_ than in the minimal I_T_-leaks model (Figure 4), consistent with a role of I_h_ in stabilizing the oscillations. This effect of Ih is independent of the sodium spiking mechanism (Figure 4, blue and gray bar; see Discussion).

## Discussion

Thalamocortical neurons have two regimes of excitability: (1) the tonic firing mode that occurs at membrane potentials positive to about −60 mV and characterized by firing of action potentials at frequencies that correlates linearly with the strength of the stimuli (McCormick and Feeser, 1990); and (2) the firing of stereotyped bursts of action potentials at high frequency at membrane potentials negative to about −65 mV (Jahnsen and Llinas, 1984). Tonic firing is said to be a relay mode that allows information to be reliably transmitted to the targeted cortical areas (i.e., TC neurons under tonic mode function as integrators) and consistently occurs during active cognitive states. The role of burst firing is less clear; it has been proposed that TC neuron bursting could play a role in stimulus detectability and/or in switching the cortical targets from inattentive states to the activated states that characterize focused attention (Weyand et al., 2001; Ortuno et al., 2014). On the other hand, periodic bursting of TC neurons correlates with brain states characterized behaviorally by cognitive arrest (deep sleep, absence seizures), and physiologically, by global synchronization of the thalamocortical system (i.e., TC neurons under repetitive burst firing mode function as resonators).

In an early study by Rush and Rinzel (1994), the bimodal excitability of TC neurons was explained by the co-existence of two oscillatory systems that operate independently at different membrane potentials and at different temporal scales. A fast system, composed by the fast amplifying sodium conductance and the fast resonant potassium conductances, operates at depolarized potentials and underlies tonic firing, whereas a slow system, composed by the gating variables (fast amplifying activation and slow resonant inactivation) of I_T_, operates at hyperpolarized membrane potentials and underlies LTS. At the most depolarized phase of these LTSs, the fast system is activated generating the characteristic burst of action potentials. It has been shown, both experimentally with TTX (see Figure 10 in McCormick and Pape, 1990) and computationally (see Figure 2C in Rush and Rinzel, 1994), that the oscillatory behavior of TC neurons is unaffected by the sodium spikes (see inset in Figure 4). These evidences support the idea that the two oscillatory systems can be studied separately and that the fast system does not affect the behavior of the slow system. Indeed, we compared the response to current injection of the minimal I_T_-leaks model with and without spiking mechanisms (HH-like models of fast Na+ and K+ currents taken from Traub et al., 2003 and implemented as in Amarillo et al., 2014), and found no differences in the range of *I_inj_* that elicit oscillations (from −6 to +2 pA; compare the range of *I_inj_* for limit cycle—gray dots in Figure 4 with the range of *I_inj_* that elicit oscillations when spiking mechanisms are present—gray bar above the bifurcation diagram in Figure 4). The only difference between the two sets of simulations is the presence of fast bursts of Na^+^-K^+^ spikes riding on the LTSs that reached the spike threshold when spiking mechanisms are present. Furthermore, we made a similar comparison with and without spiking mechanisms in the I_T_-I_h_-leaks model, and the range of *I_inj_* that elicit oscillations does not change (from −2 to −31 pA; compare the range of *I_inj_* for limit cycle—blue dots in Figure 4 with the range of *I_inj_* that elicit oscillations when spiking mechanisms are present—blue bar above the bifurcation diagram on Figure 4).

### Neurocomputational properties of the minimal I_T_-leaks model

In the present study, we focus on the dynamic structure of the slow system (given by the gating variables of I_T_) that underlies repetitive burst firing of TC neurons. The bifurcation structure—and the upper bifurcation in particular—of the minimal model that supports this slow system is consistent with the structure of a resonator, as it has been previously suggested (Hutcheon et al., 1994). The supercritical Hopf bifurcation occurring as the system enters a limit cycle from depolarized potentials enables oscillations of graded amplitude. Thus, minor hyperpolarizations induce oscillations of low amplitude without the requirement of a threshold potential (in contrast to the full blown oscillations characteristic of integrators, which usually require a large displacement of membrane potential to reach a threshold). These low amplitude oscillations could be potentiated by synaptic inputs arriving at the same frequency (resonant frequency), eventually reaching the threshold of the fast oscillatory system. The consequent action potential firing leads to neurotransmitter release, and therefore, to inter-neuronal communication, thereby promoting the synchronization of the thalamocortical system. We propose that TC neurons could anomalously enter the repetitive burst firing mode via this supercritical Hopf bifurcation during activated (depolarized) states. This would result in the pathological synchronization of the thalamocortical system at delta frequencies during wakefulness, which correlates with the occurrence of episodes of absence epilepsy.

Our analysis also shows that the behavior of the transition from a stable equilibrium to the limit cycle at hyperpolarized membrane potentials is much more complex. Depending on the value of the maximal I_T_ permeability, the system can undergo either a subcritical Hopf bifurcation or a saddle-node on invariant cycle bifurcation. This means that the neurocomputational behavior of the system can change from a resonator to an integrator, and, significantly, that this switch is controlled by the level of maximal I_T_ permeability. In addition, the time scale of the resonator increases continuously as the Bogdanov-Takens co-dimension 2 bifurcation is approached (Izhikevich, 2005).

This flexibility could have some interesting consequences on the functionality of TC cells, since resonators and integrators are driven by different optimal stimuli. Integrators fire in response to scale-free depolarizing stimuli (i.e., stimuli whose time scale depends on the firing rate of the neuron but does not depend on times scales of the intrinsic dynamics). Resonators, instead, are most efficiently driven by input stimuli containing both depolarizing and hyperpolarizing phases, with significant power in the frequency band corresponding to the intrinsic frequencies of the cell (Mato and Samengo, 2008). Choosing the adequate value of the I_T_ permeability would allow the system to control its sensitivity to inputs in the delta band or in the infra-slow band (Crunelli and Hughes, 2010) (by approaching the Bogdanov-Takens bifurcation). These properties could be very important in situations where bursting is not perfectly periodic. In Samengo et al. (2013) it was shown that, given the adequate level of variability, bursters are able to codify input information and that the coding mechanism is essentially determined by the bifurcation structure.

The other important consequence of the transition from resonator to integrator lies in the collective network behavior. Both types of neuronal behavior tend to have very different synchronization properties. Resonators tend to synchronize when these neurons interact with chemical excitatory interactions that have a time constant shorter than the period of oscillation; whereas the same interaction has a desynchronizing effect on networks of integrators (Hansel et al., 1995). For inhibition, the relation is the inverse, at least for not very strong values of the coupling constant.

I_T_ permeability can also affect network dynamics via its effect on gap junctions. This type of intercellular communication has been found in thalamocortical cells in early postnatal stages (Lee et al., 2010). The effect of gap junctions can be modulated by intrinsic currents (Pfeuty et al., 2003; Hansel et al., 2012). For instance, Pfeuty et al. (2003) showed that changing the values of sodium and potassium conductances allows to control the degree of network synchrony, from fully synchronized to a completely asynchronous behavior. In Mancilla et al. (2007) it is shown that this effect can be the opposite in some cases (see Lewis and Skinner, 2012 for a discussion on the discrepancy). In any case, the modulation of I_T_ permeability could also affect developmental processes via neuronal synchronization.

In summary, there are several mechanisms that would permit to control the dynamical state of the network and the flow of information through the thalamocortical system just by regulating up or down the permeability of I_T_.

### The role of I_H_

I_h_ not only contributes to the stabilization of the RMP in TC neurons (Amarillo et al., 2014), but it also adds robustness to the low threshold oscillations by potentiating the initial phase of depolarization and also by allowing larger excursions of the membrane potential between LTSs (favoring de-inactivation of I_T_). Oscillations elicited at hyperpolarized potentials require the activation of I_h_ to provide a recovering depolarization (pacemaker potential), which adds to the depolarizing influence of a residually activated I_T_. Loss of the stabilizing effects of I_h_ alters both the robustness of the oscillations and the voltage regime at which they occur, giving rise to more readily, yet aberrant, oscillations at more depolarized potentials. This conclusion is also supported by the absence epilepsy phenotype of the I_h_ KO mice (HCN2 principal subunit; Ludwig et al., 2003), which would otherwise contradict an essential role of I_h_ in repetitive burst firing.

In addition to these effects on the rhythmic bursting behavior of TC neurons, introduction of I_h_ tends to suppress the saddle-node bifurcation that is present in the minimal I_T_-leaks model. Hence, the presence of I_h_ would favor the resonator behavior. This means that a more complex modulation would be required to switch the system to the integrator behavior: i.e., concomitant down-regulation of I_h_ and up-regulation of I_T_.

The present study contributes to unveil the complex dynamic behavior of TC neurons. Our conclusions are in line with the suggestion that these cells have the potential to perform a sophisticated role in controlling and processing the information that flows from sensory sources to the cortex (primary thalamic nuclei), and between different cortical areas via higher order thalamic nuclei.

### Conflict of interest statement

The authors declare that the research was conducted in the absence of any commercial or financial relationships that could be construed as a potential conflict of interest.
